# The VISTA Scores: Development and Internal Validation of Novel Clinical Models for Predicting Recurrence and Mortality After Ventricular Tachycardia Ablation

**DOI:** 10.3390/diagnostics16111726

**Published:** 2026-06-03

**Authors:** Laura Stanciulescu, Maria Dorobantu

**Affiliations:** 1 Department of Cardiothoracic Pathology, Faculty of Medicine, “Carol Davila” University of Medicine and Pharmacy, 050474 Bucharest, Romania; 2 Department of Medical Sciences, Romanian Academy, 010071 Bucharest, Romania

**Keywords:** ventricular tachycardia, arrhythmic recurrence, cardiovascular mortality, risk prediction score, catheter ablation

## Abstract

**Background/Objectives**: Scar-related ventricular tachycardia (VT) remains a major contributor to morbidity and mortality in patients with structural heart disease (SHD), despite advances in catheter ablation (CA). Existing risk scores are limited by their focus on procedural outcomes, restricted variable sets, and insufficient integration of arrhythmic burden. This study aimed to bridge this gap in evidence and develop and internally validate two novel, clinically applicable prediction models—the VISTA-R and VISTA-M scores—for estimating the risk of 24-month arrhythmic recurrence and mortality following VT ablation. **Methods**: We analyzed a retrospective, single-center cohort of consecutive patients undergoing radiofrequency catheter ablation (RFCA) for scar-related VT in the setting of SHD and included a comprehensive set of clinical, arrhythmic, device-related, and procedural variables. Candidate predictors were identified through univariate logistic regression and subsequently incorporated into an exhaustive combinatorial modeling framework, generating over 1000 candidate models per endpoint. Final model selection was based on discrimination, calibration, and clinical interpretability. Internal validation was performed using leave-one-out cross-validation. **Results**: The VISTA-M model, incorporating left ventricular ejection fraction (LVEF), NYHA class IV at admission, number of clinical VT morphologies, and appropriate implantable-cardioverter defibrillator (ICD) shocks, demonstrated strong discriminative performance (AUC 0.866 in-sample, 0.826 cross-validated) and a pseudo R^2^ of approximately 30%. The VISTA-R model, including history of electrical storm (ES), ICD shocks, and VT morphologies, showed moderate discrimination (AUC 0.70 in-sample, 0.63 cross-validated) with a pseudo R^2^ of approximately 12%. Both models enabled meaningful risk stratification with progressively increasing event rates across the predefined risk classes. **Conclusions**: In conclusion, the VISTA scores provide parsimonious and clinically applicable tools for a comprehensive risk stratification after VT RFCA. Mortality is primarily driven by myocardial dysfunction and heart failure severity, whereas recurrence reflects arrhythmic burden and electrical instability. External validation is warranted to confirm these findings.

## 1. Introduction

Scar-related ventricular tachycardia (VT) remains a major contributor to morbidity and mortality in patients with structural heart disease (SHD), with high rates of recurrent arrhythmia, hospitalization, and death persisting despite advances in catheter ablation (CA) techniques [1,2,3,4,5,6,7,8]. Over the past decade, increasing efforts have focused on risk stratification to better predict outcomes following VT CA, leading to the development of several clinical prediction scores. However, their applicability in contemporary high-risk populations remains limited.

Among the most widely used tools, the PAINESD score was designed to predict periprocedural acute hemodynamic decompensation (AHD) and short-term mortality risk during VT ablation. Derived from large multicenter cohorts, it incorporates markers of advanced heart failure (HF) and systemic comorbidity burden, including reduced left ventricular ejection fraction (LVEF), presence of electrical storm (ES) and an ischemic substrate. While clinically useful for procedural risk assessment, PAINESD does not address long-term arrhythmic recurrence or post-discharge outcomes [9,10].

The I-VT score was subsequently developed to stratify patients according to the risk of VT recurrence and mortality after ablation, integrating clinical and arrhythmic variables into a prognostic framework. Although it represents a step toward longitudinal risk prediction, its performance is constrained by the limited incorporation of dynamic arrhythmic burden parameters, the heterogeneity across validation cohorts and the relatively short follow-up period [11].

Another prediction model, the RIVA score, was introduced to estimate the risk of periprocedural complications and in-hospital mortality following ventricular arrhythmia ablation. This model emphasizes procedural characteristics and baseline comorbidities, identifying factors such as age, ischemic heart disease, advanced heart failure (HF) symptoms, epicardial access, and chronic kidney disease (CKD) as key determinants of adverse events. Similar to PAINESD, however, RIVA is primarily focused on short-term safety rather than long-term clinical trajectory [12].

More recently, a dedicated risk score has been proposed to identify patients at increased risk of cardiac transplantation and/or mortality following VT ablation. Derived from a cohort of patients with SHD and a relatively longer follow-up compared with earlier models (approximately 2.8 years), the Mortalities-VA score identified LVEF, age, renal dysfunction, malignancy, and amiodarone-refractory status as independent predictors of adverse outcomes. However, the model does not incorporate measures of arrhythmic burden, which represent a key determinant of post-ablation clinical trajectory. In addition, the relatively low incidence of cardiac transplantation within the studied timeframe may limit statistical power and attenuate the robustness of the observed associations [13].

Despite the availability of these validated tools, several limitations persist. First, most existing scores are designed to predict **either procedural risk or isolated outcomes**, rather than providing an integrated assessment of both **arrhythmic recurrence and mortality.** Second, they often rely predominantly on baseline clinical characteristics, with insufficient representation of quantitative arrhythmic burden, such as the number of VT episodes, morphologies, or device therapies [14]. Third, their performance is variable across different substrates and clinical scenarios, particularly in high-risk populations enriched with electrical storm (ES) or advanced HF presentations.

Consequently, accurate prediction of both short- and mid-term outcomes after VT ablation remains challenging. There is a need for clinically applicable, parsimonious models that integrate substrate severity, arrhythmic burden, and procedural factors into a unified framework capable of supporting individualized risk stratification.

In this context, we set out to develop and internally validate two novel prediction models—the **VISTA-R and VISTA-M (V**entricular **T**achycardia **I**ntegrated **S**core for **T**herapy and **A**ssessment**—Recurrence/Mortality) scores**—in order to estimate the risk of 24-month arrhythmic recurrence and mortality, respectively, following VT ablation in a high-risk cohort of patients with SHD.

## 2. Methods

### 2.1. Study Design and Analytical Framework

The study cohort used for model development was derived from a retrospective, single-center observational population of consecutive patients who underwent radiofrequency catheter ablation (RFCA) for scar-related VT at a tertiary cardiovascular referral center. All procedures performed during the predefined study period were screened for eligibility. Patients were included if they underwent RFCA for sustained VT in the context of SHD, while those with idiopathic ventricular tachycardia, acute ischemia, primary electrical disorders, toxic cardiomyopathies, or tachycardia-induced cardiomyopathy were excluded.

SHD encompassed a broad spectrum of etiologies, including both ischemic (as defined by the presence of coronary artery disease) and non-ischemic substrates: dilated cardiomyopathy (DCM), arrhythmogenic right ventricular cardiomyopathy (ARVC), myocarditis, amyloidosis, non-dilated left ventricular cardiomyopathy (NDLVC), and primary valvular heart disease, all defined according to contemporary European guideline criteria [15,16].

ES was defined as the occurrence of three or more ventricular tachyarrhythmia episodes requiring appropriate implantable cardioverter-defibrillator (ICD) therapy within a 24 h period, or as incessant VT lasting longer than 12 h [17]. Each patient was included only once, at the time of their first ablation procedure.

The analytical framework was designed to maximize the use of available data while preserving model interpretability and ensuring applicability in clinical practice.

The initial dataset included a broad range of variables, encompassing baseline clinical characteristics, comorbidities, arrhythmic features, and procedural parameters, as detailed in the corresponding sections of this article. All available variables collected before the index ablation procedure were considered as candidate predictors during the model development process.

The study protocol adhered to the principles of the Declaration of Helsinki and received approval from the local institutional review board. Informed consent was obtained from all subjects involved in the study.

### 2.2. Candidate Variable Exploration and Preselection

An initial exploratory step consisted of performing univariate logistic regression analyses to examine the relationship between each individual variable and the outcomes of interest, namely arrhythmic recurrence and all-cause mortality at 24 months. Follow-up was complete for all the patients included in this analysis.

Due to the sensitivity of the clinical data and to avoid introducing additional assumptions or potential bias through imputation, no imputation techniques were applied. The analyses were conducted using a complete-case approach, meaning that only patients with complete information for the variables included in each model were used. The extent of missingness for each candidate predictor has been reported to ensure transparency and reproducibility.

Instead of limiting the analysis to a narrow, predefined subset of predictors, a comprehensive pool of candidate variables was maintained in order to capture the complex and multidimensional nature of the VT substrate.

This broad set of variables included baseline clinical characteristics and comorbid conditions such as age, sex, smoking status, hypertension, dyslipidemia, atrial fibrillation, type II diabetes mellitus, chronic kidney disease, the underlying substrate (ischemic versus non-ischemic), prior cardiovascular interventions, and ongoing antiarrhythmic drug therapy. Markers reflecting the severity of HF were also incorporated, specifically New York Heart Association (NYHA) functional class at admission and discharge, as well as LVEF.

In addition, detailed parameters describing arrhythmic burden were evaluated, including a history of ES, the number of clinical VT morphologies and episodes, the rate of the clinical VT, and hemodynamic stability during VT. Device-related variables were also considered, namely the presence of an ICD and the occurrence of device therapies, including both internal shocks and antitachycardia pacing (ATP), all of them before the index ablation procedure.

Finally, procedural characteristics were analyzed, encompassing the number of ablation procedures performed, the ablation approach, total procedure duration, intraprocedural VT exit sites (both their number and anatomical location), ablation targets identified during the procedure (again considering number and location), the number of involved segments according to the American Heart Association (AHA) 17-segment model, and the occurrence of periprocedural complications, classified as minor or major. Variables that demonstrated statistical significance and/or strong clinical plausibility were subsequently selected for inclusion in the multivariable modeling stage.

### 2.3. Model Development

To determine the most informative set of predictors, a fully exhaustive combinatorial strategy was applied. This involved generating and testing every possible combination of two, three, four, and five variables drawn from the candidate pool, yielding a total of 1012 distinct models for each studied endpoint. Each combination was used to construct a separate logistic regression model.

The performance of every model was evaluated using a broad range of complementary metrics. These included the area under the receiver operating characteristic curve (AUC) to quantify discriminative ability, the Brier score to assess overall prediction accuracy, and pseudo R-squared as an indicator of explained variability. In addition, the statistical significance of each predictor within the model was examined, and inter-variable correlations were assessed to identify potential multicollinearity issues.

All models were then systematically compared and ranked according to their overall performance. Particular attention was given to achieving an optimal balance between predictive accuracy, model stability, and parsimony, ensuring that the final selected models remained both robust and clinically practical.

### 2.4. Final Model Selection

The final models were chosen by integrating statistical performance with clinical interpretability, with a clear preference for parsimonious solutions that preserved strong predictive power while remaining practical for everyday clinical use.

The model developed for mortality prediction (VISTA-M) incorporated LVEF, the number of appropriate internal shocks, the number of clinical VT morphologies, and the presence of NYHA class IV symptoms at admission.

In parallel, the model designed to predict arrhythmic recurrence (VISTA-R) included a history of ES, the number of appropriate internal shocks, and the number of clinical VT morphologies.

These variables emerged consistently among the highest-performing model combinations and showed stable and clinically meaningful associations with their respective outcomes.

### 2.5. Model Validation, Performance Assessment and Risk Stratification

Internal validation of the model was carried out using a leave-one-out cross-validation (LOOCV) strategy. In this approach, each individual observation is excluded once from the dataset, and the model is trained on all the remaining data points. The trained model is then used to generate a prediction for the excluded observation. This process is repeated for every observation in the dataset, so that each data point serves as a test case exactly once. By systematically rotating the left-out observation, this method provides a rigorous and nearly unbiased estimate of how the model is expected to perform on new, unseen data, thereby offering a robust assessment of its out-of-sample predictive ability.

Model performance was assessed across several complementary dimensions to ensure a comprehensive evaluation. Discrimination, which reflects the model’s ability to correctly distinguish between individuals who experience the event and those who do not, was quantified using the area under the receiver operating characteristic curve (AUC). This metric was calculated both on the original training data (in-sample) and within the cross-validation framework to evaluate how well the model generalizes beyond the data it was trained on.

Calibration was examined by comparing the probabilities predicted by the model with the actual observed event rates. This step assesses whether the predicted risks are numerically accurate, meaning that, for example, a predicted probability of 20% truly corresponds to an observed event rate of approximately 20% in similar individuals.

Finally, model separation was evaluated by analyzing the distribution of predicted probabilities in patients who experienced the event versus those who did not. This analysis provides insight into how distinctly the model assigns higher risk scores to event cases compared to non-event cases, reflecting its practical usefulness in stratifying patients according to risk.

Predicted probabilities generated by the final models were further translated into clinically relevant risk thresholds, allowing patients to be grouped into clearly defined risk categories. This transformation from continuous probabilities to discrete categories enhances practical usability, as it aligns model outputs with decision-making processes commonly used in clinical settings.

To support interpretation and facilitate real-world application, these risk categories were then presented through dedicated visualizations. These graphical representations were designed to clearly display how patients are distributed across risk groups and to highlight differences in outcomes between them, thereby making the results more accessible and meaningful for clinicians.

## 3. Results

### 3.1. Patient Characteristics

We retrospectively included 142 consecutive patients (63 ± 14 years; 80.28% male) presenting with at least one episode of SMVT requiring a first catheter ablation procedure.

Within the overall population, ischemic and non-ischemic substrates were almost equally represented (57.7% and 42.3%, respectively) and patients with ICM were slightly older (66.94 ± 10.42 vs. 58.1 ± 17.13 years, *p* < 0.001).

Among non-ischemic substrates, DCM was the most common phenotype (33.33%, *n* = 20), followed by ARVC (26.66%, *n* = 16). These phenotypes were also most frequently associated with ES at presentation (14 patients with DCM and 9 with ARVC).

The baseline characteristics for the study population are summarized in Table 1.

### 3.2. Procedural Characteristics

Procedural characteristics were broadly comparable between groups. Patients with ICM exhibited a numerically higher number of clinical VT episodes (17.6 ± 22.6 vs. 13.36 ± 25.57, *p* = 0.31), although this difference did not reach statistical significance. Similarly, the number of clinical VT morphologies (1.11 ± 0.31 vs. 1.25 ± 0.62, *p* = 0.13) and inducible VT morphologies during the procedure (2.13 ± 1.96 vs. 2.03 ± 1.47, *p* = 0.71) were comparable between ischemic and non-ischemic groups. The number of RFCA procedures performed per patient was also similar (1.31 ± 0.73 vs. 1.5 ± 0.98, *p* = 0.20). In contrast, procedural duration was significantly longer in patients with NICM (247.51 ± 90.11 vs. 206.24 ± 51.03, *p* = 0.002). The complete procedural characteristics of the study population are displayed in Table 2.

### 3.3. VISTA-M (Mortality Prediction Model)

**Figure 1:**
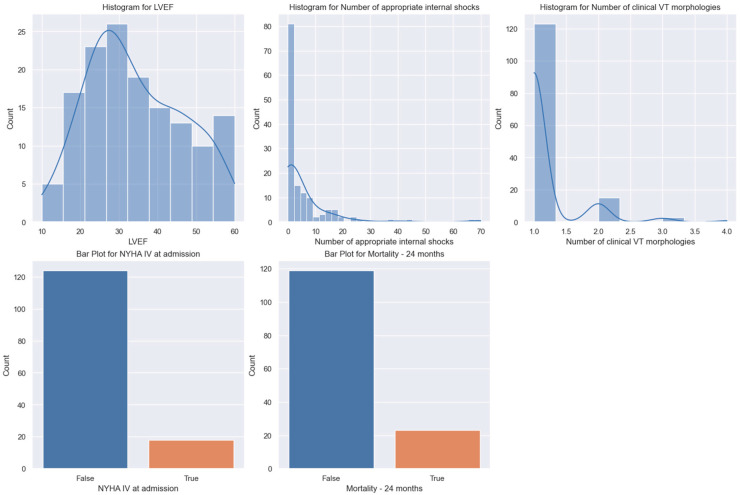
Depiction of the distribution of the continuous variables, along with the frequency of NYHA class IV status at admission and the counts of the target variable—mortality at 24 months.

In the mortality model, the distribution of predictor variables demonstrated that most patients had an LVEF between 20% and 40%, with only a small fraction showing more extreme values (<10% or >50%). The number of appropriate ICD shocks was generally low, with most patients having values close to zero, while higher counts (>20) were less frequent (Figure 1).

**Figure 2:**
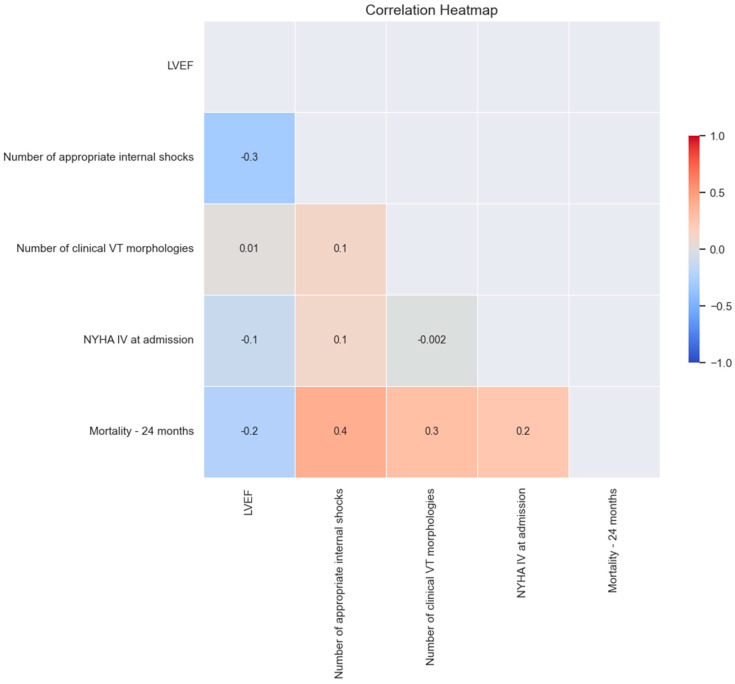
Pearson correlation matrix for the mortality prediction model.

Evaluation of the correlation matrix showed no meaningful relationships between the independent variables, suggesting a minimal likelihood of multicollinearity. At the same time, modest correlations were identified between the predictors and the outcome variable, providing further justification for their inclusion in the multivariable model (Figure 2).

The final logistic regression model (VISTA-M) included LVEF, number of appropriate internal shocks, number of clinical VT morphologies, and NYHA class IV at admission. All model coefficients were statistically significant (*p* < 0.05). The exact calculation method is displayed below:

Z = β_0_ + β_1_·LVEF + β_2_·Number of appropriate internal shocks + β_3_·Number of clinical VT morphologies + β_4_·NYHA IV at admission

β̂_0_ = −3.0370

β̂_1_ = −0.0600 (LVEF)

β̂_2_ = 0.0918 (Number of appropriate internal shocks)

β̂_3_ = 1.8309 (Number of clinical VT morphologies)

β̂_4_ = 1.8011 (NYHA IV at admission)

**Figure 3:**
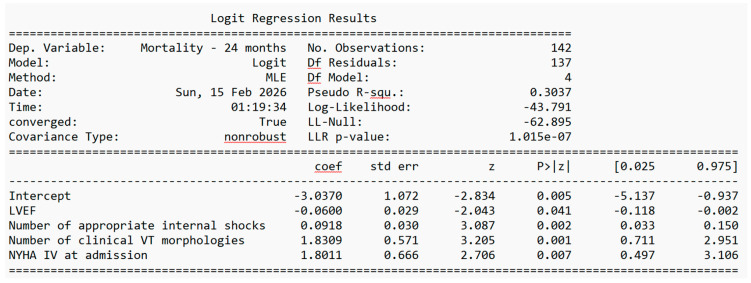
Logit regression results for the mortality prediction model.

The pseudo R-squared value is approximately 30%, suggesting that the model accounts for a relevant portion of the variability in 24-month mortality. However, a considerable proportion—around 70%—remains attributable to factors not captured within the model (Figure 3).

The VISTA-M model exhibited strong discriminative ability, achieving an AUC of 0.866 in the training dataset and 0.826 after leave-one-out cross-validation. Both values were highly significant when compared with the null hypothesis of no discriminative capacity (AUC = 0.5, *p* < 0.000001), indicating that the model performs markedly better than chance. The small reduction in AUC between the in-sample and cross-validated analyses further underscores the model’s stability and its capacity to generalize to new data.

**Figure 4:**
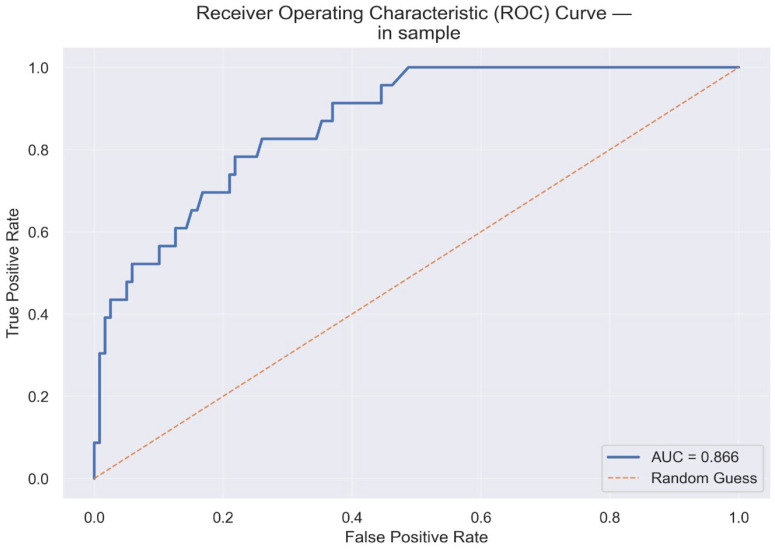
ROC curve for the in-sample analysis for the mortality model.

**Figure 5:**
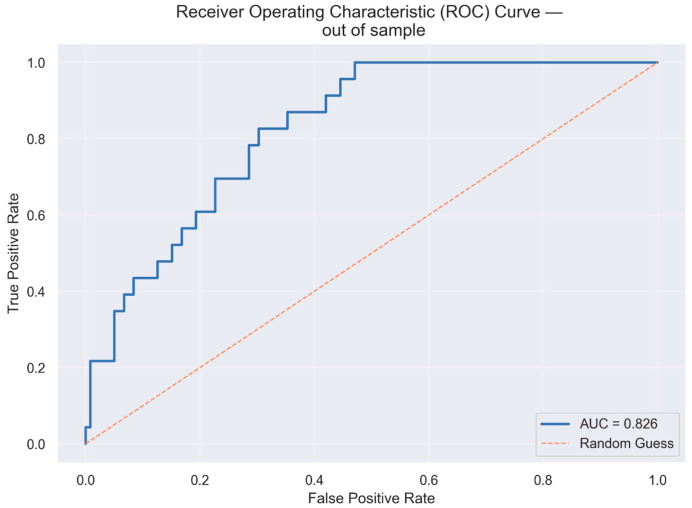
ROC curve for the out-of-sample analysis for the mortality model.

The discriminative performance of the VISTA-M model is depicted in Figure 4 and Figure 5, which display the receiver operating characteristic (ROC) curves for both in-sample and cross-validated settings. The substantial overlap between these curves reflects consistent performance across datasets and supports the model’s potential external applicability. However, considering the model’s characteristics and the single-center retrospective design, external validation in independent multicenter cohorts would be required before any clinical implementation is attempted.

**Figure 6:**
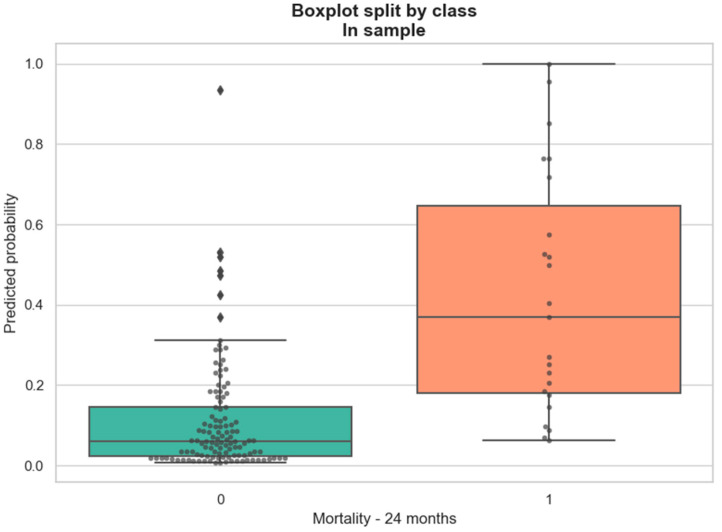
Boxplot stratified by class (event vs. non-event) for in-sample predictions of the mortality prediction model.

**Figure 7:**
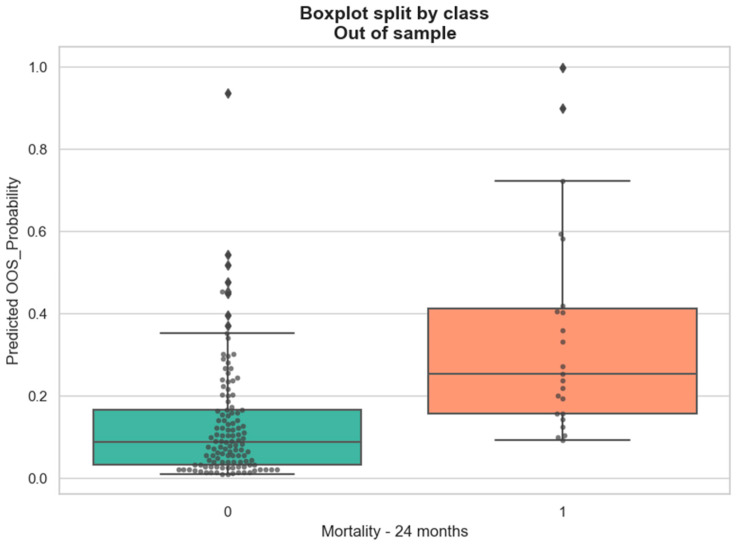
Boxplot stratified by class (event vs. non-event) for out-of-sample predictions of the mortality prediction model.

Additional insight into model performance is illustrated in Figure 6 and Figure 7, which present the distribution of predicted probabilities stratified by outcome (event versus non-event) for both in-sample and cross-validated predictions. Patients who did not experience mortality generally show lower predicted probabilities, whereas higher values are predominantly observed among those with events, indicating effective separation between the two groups. The comparable shape and spread of these distributions across training and validation analyses further reinforce the model’s consistency and robustness.

**Figure 8:**
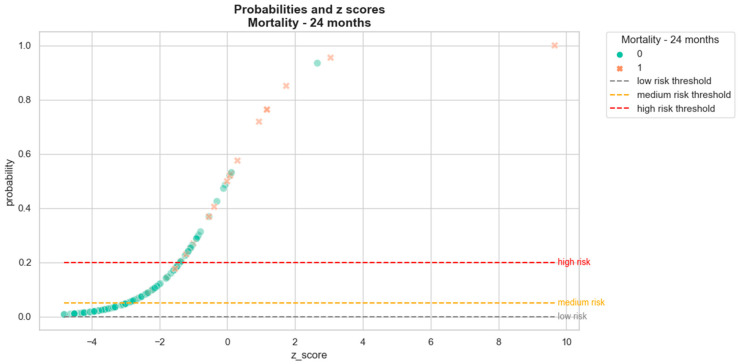
Probabilities and z scores for mortality at 24 months.

Risk stratification based on predicted probabilities is illustrated in Figure 8, which shows the transformation of the latent logistic score into probability and emphasizes the fundamental mechanism of the proposed calculator. The logistic regression model produces a latent score (z), which is subsequently transformed into a probability through the logistic function:

$$P(z)=\;\frac{1}{1+e^{-z}}$$

where e = Euler’s constant.

Using this transformation, patients were categorized into three clinically meaningful risk strata—low, intermediate, and high—corresponding to progressively increasing predicted probabilities of mortality.

As illustrated, the incidence of mortality rises stepwise across these categories. No events were recorded in the low-risk group (0–0.05; 0/51), while the intermediate-risk group (0.05–0.2) accounted for 7 events among 54 patients (event rate 12.96%). In contrast, the high-risk group (>0.2) included 16 events among 37 patients, corresponding to an event rate of 43.24% (Table 3). These results support the model’s ability to effectively discriminate between patients with differing levels of mortality risk. The proposed risk thresholds should presently be regarded as preliminary and will require confirmation and potential recalibration through prospective external validation in independent multicenter cohorts.

### 3.4. VISTA-R (Recurrence Prediction Model)

**Figure 9:**
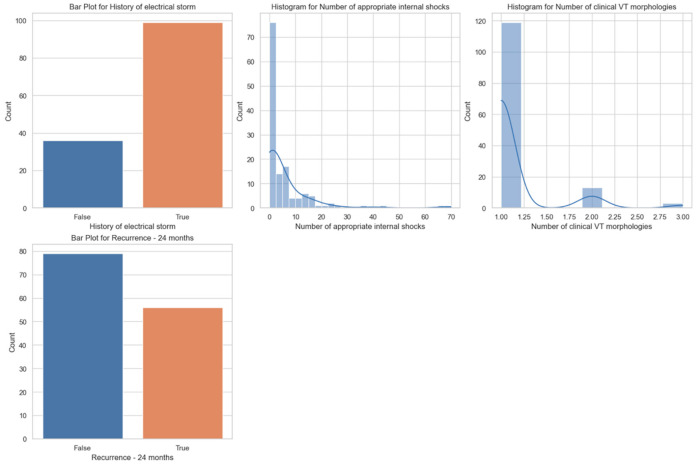
Depiction of the distribution of the continuous variables, along with the history of ES and the counts of the target variable—arrhythmic recurrence at 24 months.

The distribution of variables within the recurrence model showed a different pattern compared with the mortality model, characterized by a more even distribution of outcomes between patients with and without events. A history of electrical storm was observed in most patients, while the majority presented with a single clinical ventricular tachycardia morphology, and progressively fewer individuals exhibited multiple morphologies (Figure 9).

**Figure 10:**
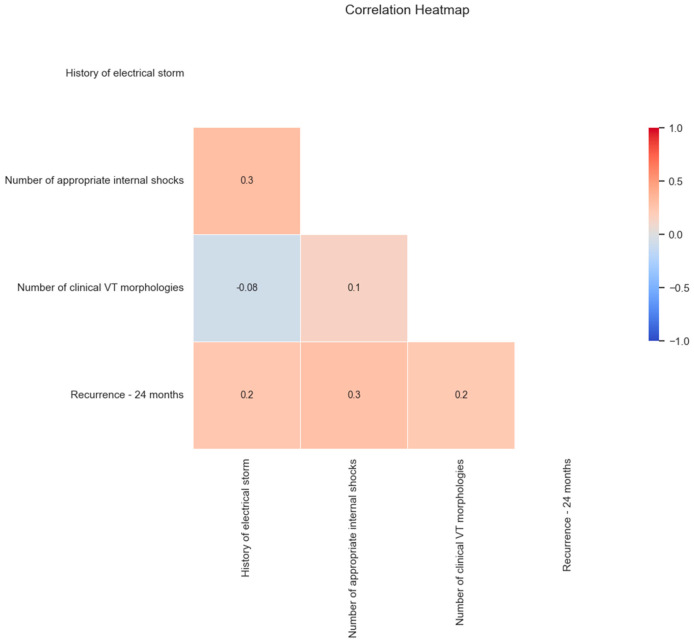
Pearson correlation matrix for the arrhythmic recurrence prediction model.

Assessment of the correlation matrix revealed no significant relationships among the independent variables, suggesting a minimal risk of multicollinearity. Concurrently, modest yet meaningful associations were observed between the predictors and arrhythmic recurrence, supporting their relevance and inclusion in the model (Figure 10).

**Figure 11:**
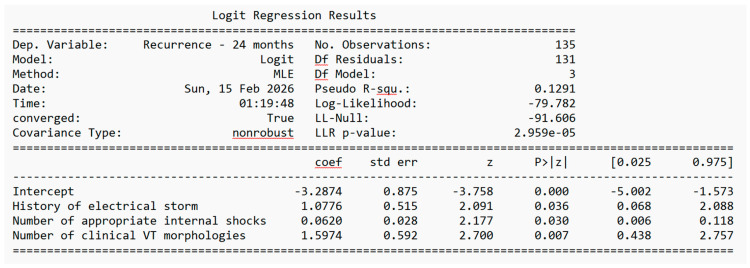
Logit regression results for the arrhythmic recurrence prediction model.

The final VISTA-R model comprised three predictors: history of ES, number of appropriate internal shocks, and number of clinical VT morphologies. Each of these variables reached statistical significance (*p* < 0.05), supporting their independent role in predicting arrhythmic recurrence. The pseudo R-squared value was approximately 12%, indicating that the model accounts for a meaningful, though limited, proportion of the variability in 24-month recurrence, with the majority (around 88%) likely explained by factors not included in the model (Figure 11). The final model specification is presented below:

Z = β_0_ + β_1_· History of electrical storm + β_2_·Number of appropriate internal shocks + β_3_·Number of clinical VT morphologies

β̂_0_ = −3.2874

β̂_1_ = 1.0776 (History of electrical storm)

β̂_2_ = 0.062 (Number of appropriate internal shocks)

β̂_3_ = 1.5974 (Number of clinical VT morphologies)

**Figure 12:**
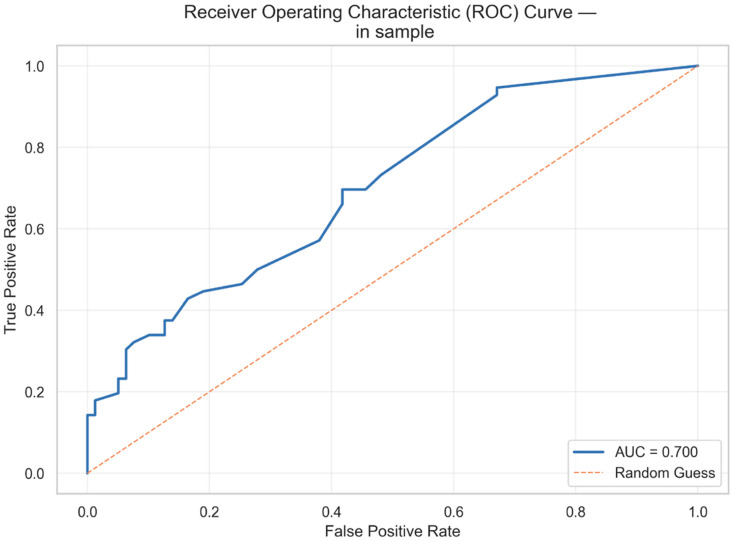
Boxplot stratified by class (event vs. non-event) for in-sample predictions for the arrhythmic recurrence model.

**Figure 13:**
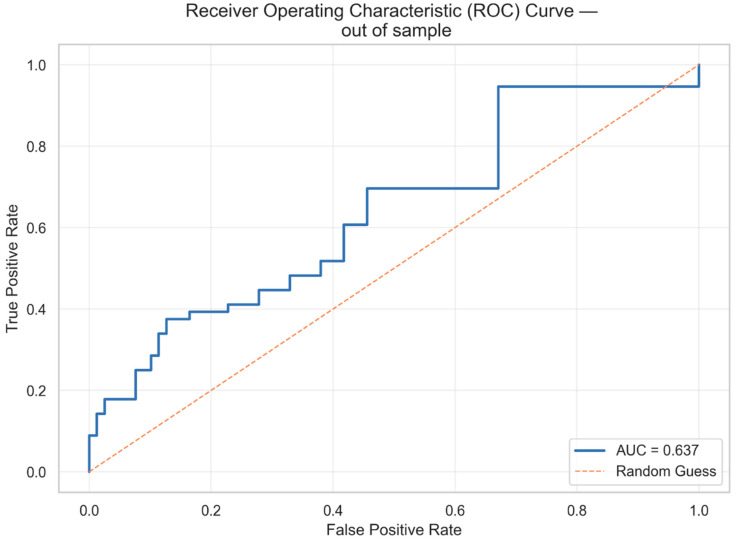
ROC curve for the out-of-sample analysis for the arrhythmic recurrence model.

The VISTA-R model showed moderate discriminative ability, with an AUC of 0.70 in the training dataset and 0.63 after leave-one-out cross-validation. Both estimates were statistically significant relative to the null hypothesis of no discrimination (AUC = 0.5), indicating performance above chance. The reduction in AUC observed in the cross-validated analysis points to a modest decline in performance when the model is applied to new data, likely reflecting the complex and multifactorial nature of arrhythmic recurrence. Overall, these results suggest that the model captures a relevant predictive signal, although its discriminative power remains limited, supporting its use primarily as an exploratory tool for risk stratification rather than a definitive predictive model. Model discrimination is illustrated in Figure 12 and Figure 13.

**Figure 14:**
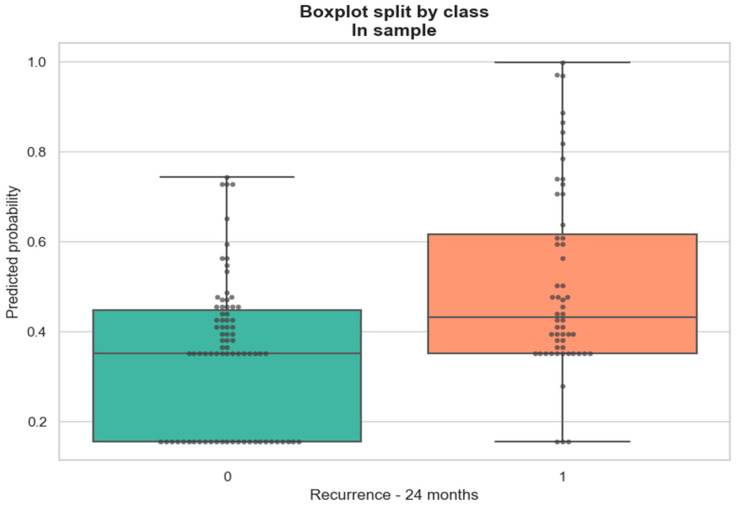
Distribution of predicted probabilities (in-sample) according to recurrence status.

**Figure 15:**
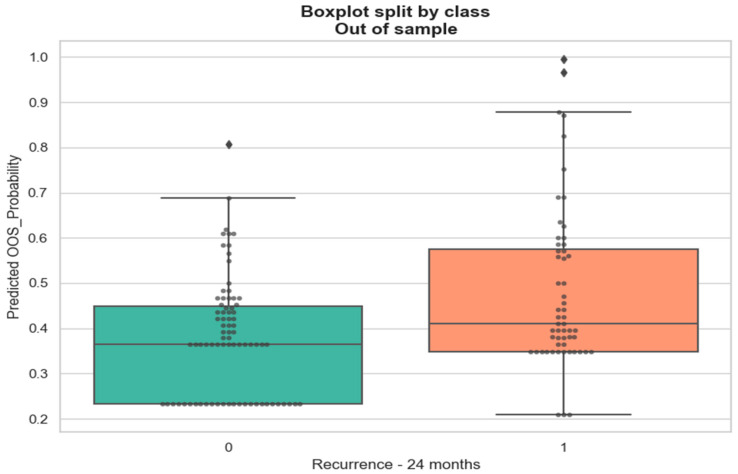
Distribution of predicted probabilities (out-of-sample) according to recurrence status.

Further evaluation of model performance is illustrated in Figure 14 and Figure 15, which show the distribution of predicted probabilities stratified by outcome (recurrence versus no recurrence). Lower probability values are mainly observed among patients without recurrence, while higher predicted risks are more frequent in those who experienced events. The comparable distributions between the in-sample and cross-validated analyses provide additional evidence of the model’s stability.

**Figure 16:**
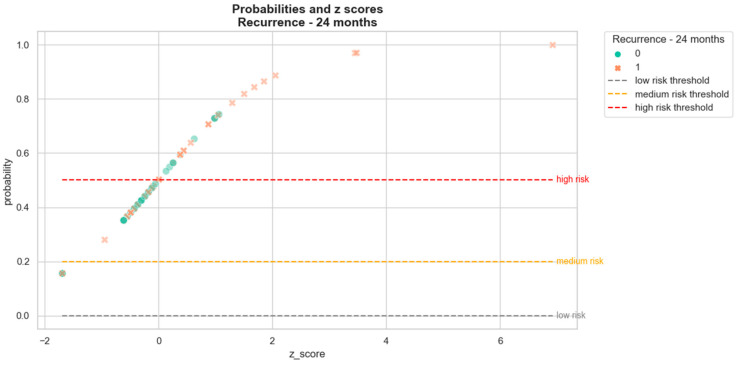
Risk stratification based on predicted probabilities using the VISTA-R model.

Risk stratification based on predicted probabilities is presented in Figure 16, demonstrating the transformation of the underlying logistic score into probability estimates. Applying the same approach used for the mortality model, patients were categorized into three distinct risk groups. A clear, stepwise increase in recurrence rates was observed across these categories (Table 4).

In the low-risk group (0–0.2), 3 events were recorded among 29 patients, corresponding to an event rate of 10.34%. The intermediate-risk category (0.2–0.5) included 32 events among 75 patients (event rate 42.67%), while the high-risk group (>0.5) comprised 21 events among 31 patients, yielding an event rate of 67.74%. These findings highlight the model’s capacity to meaningfully stratify patients according to their risk of arrhythmic recurrence. Given the complex and multifactorial nature of post-ablation arrhythmic recurrence, these stratification thresholds should currently be interpreted with caution and will require prospective validation and refinement in larger independent multicenter populations.

## 4. Discussions and Limitations

Our analysis outlines the development and internal validation of two concise and clinically applicable prediction models—the VISTA scores—designed to estimate the risk of arrhythmic recurrence and mortality following VT ablation in patients with structural heart disease. Several key findings arise from these results.

First, the data emphasize the differing predictability of mortality versus arrhythmic recurrence in this patient population. The mortality model (VISTA-M) demonstrated excellent discriminative performance, with consistently high AUC values that remained stable between in-sample and cross-validated analyses, supporting both robustness and generalizability. In contrast, the recurrence model (VISTA-R) showed only moderate discriminative ability, with a decline in performance during out-of-sample validation, highlighting the more complex and dynamic nature of arrhythmic recurrence in these patients. Notably, both models maintained clear separation between event and non-event groups when examining predicted probability distributions and enabled reliable stratification into risk categories with progressively increasing event rates, supporting their internal validity. The absence of a marked difference in performance between training and validation analyses suggests that substantial overfitting is unlikely, despite the relatively limited cohort size.

At a predefined high-risk threshold, the VISTA-M model achieved a sensitivity of 69.6% and specificity of 82.4% for predicting 24-month mortality. In contrast, the VISTA-R model demonstrated a sensitivity of 37.5% and specificity of 87.3%, indicating higher specificity but more limited sensitivity for recurrence prediction. Taken together, these observations indicate that VISTA-M provides strong and clinically meaningful prognostic insight, whereas VISTA-R should be viewed primarily as an exploratory model capturing part of a multifactorial process.

Second, the predictors retained in the final models offer important insights into the pathophysiological mechanisms underlying outcomes after VT ablation. Mortality was mainly associated with indicators of global myocardial dysfunction and advanced heart failure, as reflected by LVEF and NYHA class IV at admission, along with markers of electrical instability such as the number of appropriate internal shocks and the complexity of VT morphologies. In contrast, recurrence appeared to be driven predominantly by arrhythmic burden and prior electrical instability, as demonstrated by the inclusion of electrical storm, ICD therapies, and VT morphologies in the VISTA-R model. These findings support the concept that mortality reflects the overall severity of the underlying myocardial disease, whereas recurrence is more closely linked to the persistence and complexity of the arrhythmogenic substrate.

Contemporary HF pharmacotherapy may further reshape the prognostic landscape in patients undergoing VT ablation. Sodium-glucose cotransporter-2 inhibitors (SGLT2i) and angiotensin receptor–neprilysin inhibition (ARNI) have consistently improved HF trajectories and may also attenuate arrhythmic vulnerability through reverse remodeling, improved loading conditions, reduced congestion, favorable metabolic effects, and modulation of neurohormonal activation. Recent device-based and meta-analytic data suggest that these therapies may reduce ventricular arrhythmias, appropriate ICD therapies, and shock burden, although the magnitude of benefit and the mechanisms involved remain incompletely defined. Consequently, broader uptake of these agents in future cohorts could modify both baseline risk profiles and event rates after VT ablation, particularly for predictors related to HF severity, ICD shocks, and arrhythmic burden [18,19,20,21,22,23,24,25,26,27,28,29,30,31]. This may influence model calibration and discrimination over time, underscoring the need for external validation and periodic recalibration of prediction tools such as VISTA-R and VISTA-M in cohorts treated according to contemporary guideline-directed medical therapy.

Furthermore, despite their relative simplicity, both models were able to effectively stratify patients into distinct risk categories, with a clear gradient of increasing event rates across groups. This has important clinical implications, suggesting that even a limited number of carefully selected variables can yield meaningful and actionable risk stratification. The consistency observed between in-sample and cross-validated predictions further reinforces the reliability of the modeling approach.

When placed in the context of existing risk stratification tools, the VISTA models address several limitations of currently available scores. Most established tools, such as PAINESD or RIVA, primarily focus on periprocedural risk or short-term outcomes, while others, including the I-VT score, provide broader prognostic assessment, while relying largely on baseline clinical variables. In contrast, the VISTA scores incorporate quantitative measures of arrhythmic burden alongside markers of disease severity, offering a more comprehensive representation of the determinants influencing post-ablation outcomes.

At the same time, the relatively modest performance of the recurrence model underscores the ongoing challenge of predicting arrhythmic events in this setting. Even with multiple indicators of electrical instability included, a significant proportion of variability remains unexplained, suggesting that additional factors—such as detailed substrate characteristics, completeness of ablation, or postprocedural remodeling—likely play an important role.

Overall, our findings support a dual-framework approach to risk stratification following VT ablation, recognizing that mortality and recurrence are driven by partially distinct mechanisms and may require separate predictive tools. The VISTA scores represent a step in this direction, providing two complementary models that capture different aspects of post-ablation risk. Thus, the proposed models should currently be interpreted as hypothesis-generating and exploratory prognostic tools rather than instruments intended for immediate bedside implementation or therapeutic decision-making.

We acknowledge several limitations that should be considered when interpreting these results. First, the study is based on a retrospective, single-center cohort, which introduces potential selection and referral bias and may limit the generalizability of the findings. Differences in patient management, procedural strategies, and follow-up protocols across centers could influence both outcomes and model performance. Second, the relatively small sample size, particularly in relation to the number of candidate variables initially evaluated, increases the risk of type II error and may affect the stability of the selected predictors. Although a structured and exhaustive model selection process was applied and internal validation was performed using leave-one-out cross-validation, residual overfitting cannot be entirely excluded. Third, the models were both developed and validated within the same dataset, without external validation in an independent cohort, meaning that the reported performance metrics represent internal estimates and may not fully translate to other populations. Fourth, the moderate performance of the recurrence model reflects the inherent complexity of arrhythmic outcomes. Key determinants such as detailed scar architecture, completeness of ablation, and post-ablation remodeling were not systematically captured and may account for a large portion of the unexplained variability.

Nonetheless, given the exhaustive combinatorial model selection strategy, a degree of selection bias in the reported performance estimates cannot be entirely excluded despite internal cross-validation.

In addition, temporal changes in guideline-directed HF therapy, particularly the increasing use of SGLT2 inhibitors and ARNI, were not modeled as longitudinal modifiers and may affect future event rates, predictor strength, and model transportability.

Finally, although a wide range of clinically relevant variables was included, the models are limited by the available dataset and do not incorporate advanced imaging data, invasive substrate characterization, or longitudinal dynamic variables, all of which could potentially enhance predictive accuracy.

## 5. Conclusions

Our study reports the development and internal validation of two clinically applicable prediction models (VISTA scores) for estimating arrhythmic recurrence (VISTA-R) and mortality (VISTA-M) after VT ablation in patients with SHD.

VISTA-M demonstrated strong discrimination, with mortality primarily associated with myocardial dysfunction and heart failure severity, alongside markers of electrical instability. In contrast, VISTA-R showed modest performance, reflecting the multifactorial nature of arrhythmic recurrence in these patients, driven mainly by arrhythmic burden and prior electrical instability.

Both models enabled clinically meaningful risk stratification, supporting their potential use in post-ablation risk assessment and follow-up planning. However, they should currently be regarded as hypothesis-generating and exploratory prognostic tools rather than instruments intended for immediate bedside implementation or therapeutic decision-making.

External validation in larger, prospective multicenter cohorts would be required before proper clinical implementation is attempted, in order to confirm the robustness and generalizability of these findings and to further refine model performance through the integration of additional markers of substrate complexity, heart failure progression, and disease severity into a more comprehensive risk stratification framework.

## Figures and Tables

**Table 1:** Baseline characteristics of the study population.

	All (*n* = 142)	ICM (*n* = 82)	NICM (*n* = 60)	Odds Ratio/Mean Differences (95% CI)	*p*-Value
Males	80.28% (114)	85.36% (70)	73.33% (44)	OR 2.12 (0.97–4.66)	0.06
Age	63 ± 14	66.94 ± 10.42	58.1 ± 17.13	MD 8.84 (3.89–13.79)	<0.001
Hypertension	68.31% (97)	84.14% (69)	46.66% (28)	OR 6.06 (2.93–12.54)	<0.001
T2DM	23.94% (34)	26.83% (22)	20% (12)	OR 1.47 (0.68–3.19)	0.35
CKD	27.46% (39)	35.36% (29)	16.66% (10)	OR 2.74 (1.25–5.99)	0.01
Dyslipidemia	73.24% (104)	93.9% (77)	45% (27)	OR 18.81 (6.87–51.49)	<0.001
History of AF	32.4% (46)	31.7% (26)	33.33% (20)	OR 0.93 (0.47–1.82)	0.84
History of ES	72.53% (103)	79.26% (65)	63.33% (38)	OR 2.21 (1.10–4.44)	0.025
Active smoker	23.24% (33)	34.14% (28)	8.33% (5)	OR 5.70 (2.02–16.08)	0.001
Prior PCI	44.36% (63)	71.95% (59)	6.66% (4)	OR 35.91 (11.69–110.29)	<0.001
Prior CABG	5.63% (8)	7.76% (8)	0% (0)	OR 7.03 (0.40–124.79)	0.106
Prior cardiac surgery (other than CABG)	2.11% (3)	2.44% (2)	1.66% (1)	OR 1.48 (0.13–16.82)	0.74
Amiodarone at the time of the procedure	62.67% (89)	68.3% (56)	55% (33)	OR 1.76 (0.90–3.44)	0.09
Beta-blocker at the time of the procedure	87.32% (124)	95.12% (78)	76.66% (46)	OR 5.93 (1.86–18.93)	0.002
NYHA I at admission	4.93% (7)	1.22% (1)	10% (6)	OR 0.11 (0.01–0.91)	0.04
NYHA II at admission	36.62% (52)	29.26% (24)	46.66% (28)	OR 0.48 (0.24–0.97)	0.04
NYHA III at admission	45.77% (65)	52.44% (43)	36.66% (22)	OR 1.90 (0.96–3.74)	0.06
NYHA IV at admission	12.67% (18)	17.07% (14)	6.66% (4)	OR 2.88 (0.89–9.36)	0.08
Clinical VT rate (ms)	373.76 ± 54.22	373.4 ± 46.01	374.25 ± 64.18	MD −0.85 (−19.5–17.8)	0.93
Hemodynamically stable while in VT	88.73% (126)	95.12% (78)	80% (48)	OR 4.88 (1.52–15.63)	0.007
ICD present	63.38% (90)	65.85% (54)	60% (36)	OR 1.29 (0.66–2.54)	0.45
LVEF	34.2 ± 12.26	30.35 ± 10.02	39.46 ± 13.14	MD −9.11 (−13.0–−5.2)	<0.001

**Table 2:** Procedural characteristics of the study population.

	All (*n* = 142)	ICM (*n* = 82)	NICM (*n* = 60)	Odds Ratio/Mean Differences (95% CI)	*p*-Value
Number of clinical VT episodes	15.8 ± 23.91	17.6 ± 22.6	13.36 ± 25.57	MD 4.24 (−4.01–12.49)	0.31
Number of clinical VT morphologies	1.17 ± 0.47	1.11 ± 0.31	1.25 ± 0.62	MD −0.14 (−0.32–0.04)	0.13
Number of inducible VT morphologies	2.09 ± 1.77	2.13 ± 1.96	2.03 ± 1.47	MD 0.10 (−0.46–0.66)	0.72
Clinical VT rate (ms)	373.76 ± 54.22	373.4 ± 46.01	374.25 ± 64.18	MD −0.85 (−19.5–17.8)	0.93
Number of ablation procedures	1.39 ± 0.85	1.31 ± 0.73	1.5 ± 0.98	MD −0.19 (−0.48–0.10)	0.20
Procedural duration (min)	223.68 ± 72.88	206.24 ± 51.03	247.51 ± 90.11	MD −41.27 (−67.4–−15.1)	0.002
Endocardial ablation	69.71% (99)	86.58% (71)	46.66% (28)	OR 7.39 (3.57–15.28)	<0.001
Epicardial ablation	2.11% (3)	0% (0)	5% (3)	OR 0.09 (0.00–1.72)	0.11
Endo-epicardial ablation	28.16% (40)	13.41% (11)	48.33% (29)	OR 5.99 (2.79–12.86)	<0.001
Number of involved AHA segments	3.63 ± 1.77	3.84 ± 1.74	3.23 ± 1.78	MD 0.61 (−0.03–1.25)	0.06
Presence of RV involvement	21.12% (30)	8.53% (7)	38.33% (23)	OR 0.15 (0.06–0.38)	<0.001
Acute partial procedural success	88.73% (126)	92.68% (76)	83.33% (50)	OR 2.53 (0.85–7.49)	0.09
Acute complete procedural success	75.35% (107)	84.14% (69)	63.33% (38)	OR 3.07 (1.50–6.27)	0.002

**Table 3:** Number of events and event rate for each risk group within the mortality prediction model.

Risk Group	Interval	*n*	Events	Event Rate
Low risk	[0, 0.05]	51	0	0
Intermediate risk	[0.05, 0.2]	54	7	0.12963
High risk	[0.2, 1]	37	16	0.432432

**Table 4:** Number of events and event rate for each risk group within the arrhythmic recurrence prediction model.

Risk Group	Interval	*n*	Events	Event Rate
Low risk	[0, 0.2]	29	3	0.103448
Intermediate risk	[0.2, 0.5]	75	32	0.426667
High risk	[0.5, 1]	31	21	0.677419

## Data Availability

The original contributions presented in this study are included in the article. Further inquiries can be directed to the corresponding author.

## Abbreviations

The following abbreviations are used in this manuscript:

VT	Ventricular Tachycardia
SHD	Structural Heart Disease
CA	Catheter Ablation
VISTA-R	Ventricular Tachycardia Integrated Score for Therapy and Assessment-Recurrence
VISTA-M	Ventricular Tachycardia Integrated Score for Therapy and Assessment-Mortality
RFCA	Radiofrequency Catheter Ablation
LVEF	Left Ventricular Ejection Fraction
NYHA	New York Heart Association
ICD	Implantable Cardioverter-Defibrillator
AUC	Area Under the Curve
ES	Electrical Storm
I-VT	Ventricular Tachycardia Score
RIVA	Risk in Ventricular Ablation
HF	Heart Failure
CKD	Chronic Kidney Disease
DCM	Dilated Cardiomyopathy
ARVC	Arrhythmogenic Right Ventricular Cardiomyopathy
NDLVC	Non-Dilated Left Ventricular Cardiomyopathy
AHA	American Heart Association
ATP	Antitachycardia Pacing
LOOCV	Leave-One-Out Cross-Validation
ROC	Receiver Operating Characteristic
CI	Confidence Interval
OR	Odds Ratio
MD	Mean Difference
ICM	Ischemic Cardiomyopathy
NICM	Non-Ischemic Cardiomyopathy
T2DM	Type 2 Diabetes Mellitus
AF	Atrial Fibrillation
PCI	Percutaneous Coronary Intervention
CABG	Coronary Artery Bypass Grafting
RV	Right Ventricle
SMVT	Sustained Monomorphic Ventricular Tachycardia
